# Supplementary figures and images for: Exploiting DNA methylation in cassava under water deficit for crop improvement
Source: PLoS One. 2024 Feb 22;19(2):e0296254. doi: 10.1371/journal.pone.0296254 (PMC10883565; doi:10.1371/journal.pone.0296254)

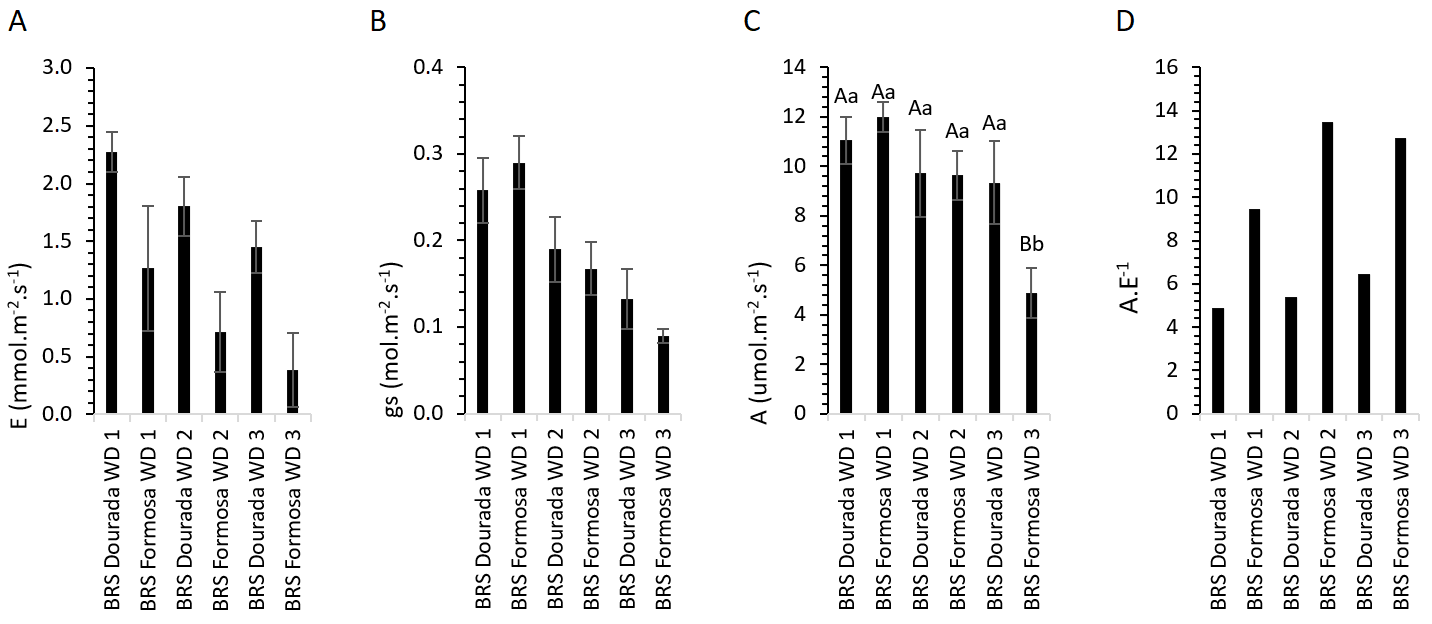

Supplement: S1 Fig — (TIF) [file pone.0296254.s001.tif]

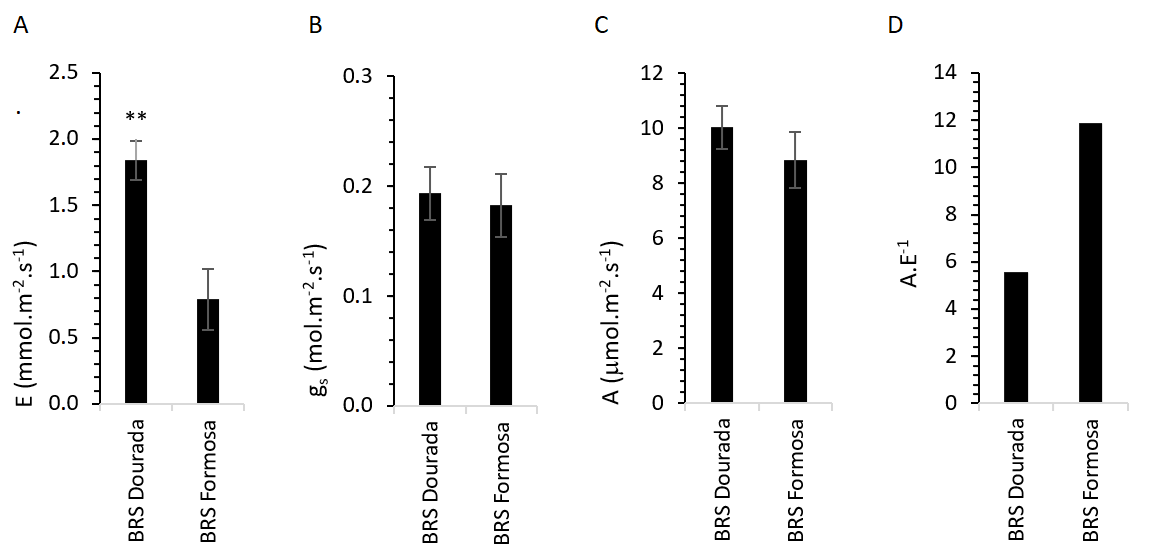

Supplement: S2 Fig — (TIF) [file pone.0296254.s002.tif]

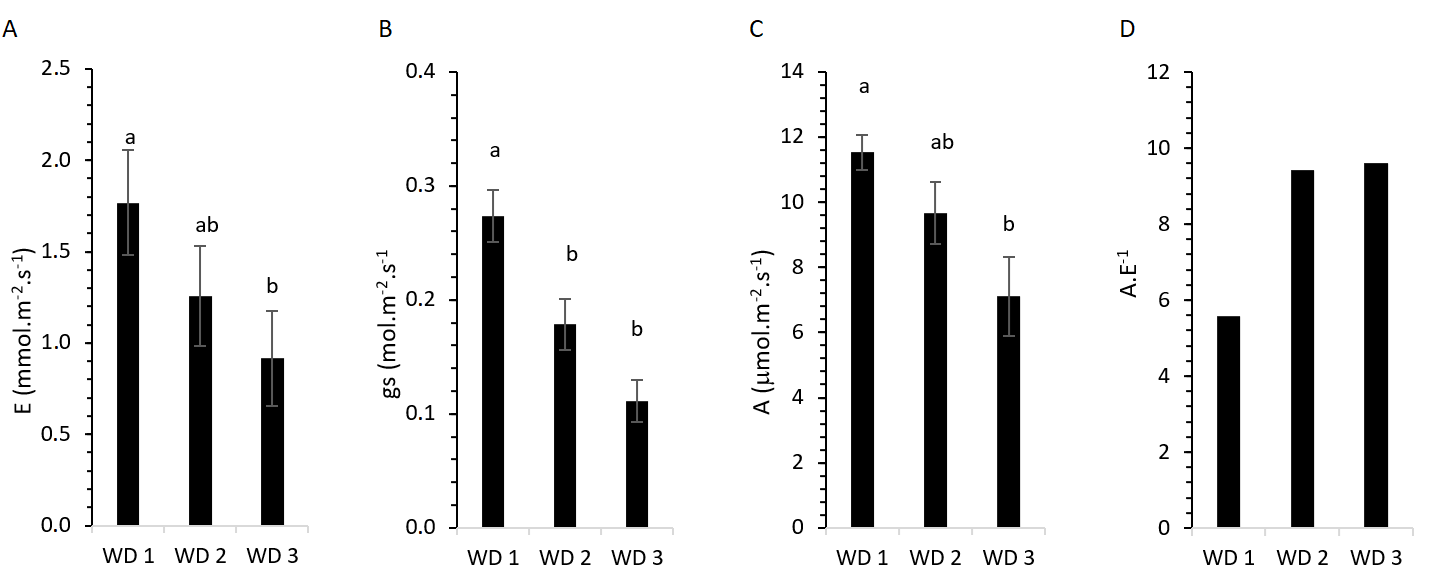

Supplement: S3 Fig — (TIF) [file pone.0296254.s003.tif]

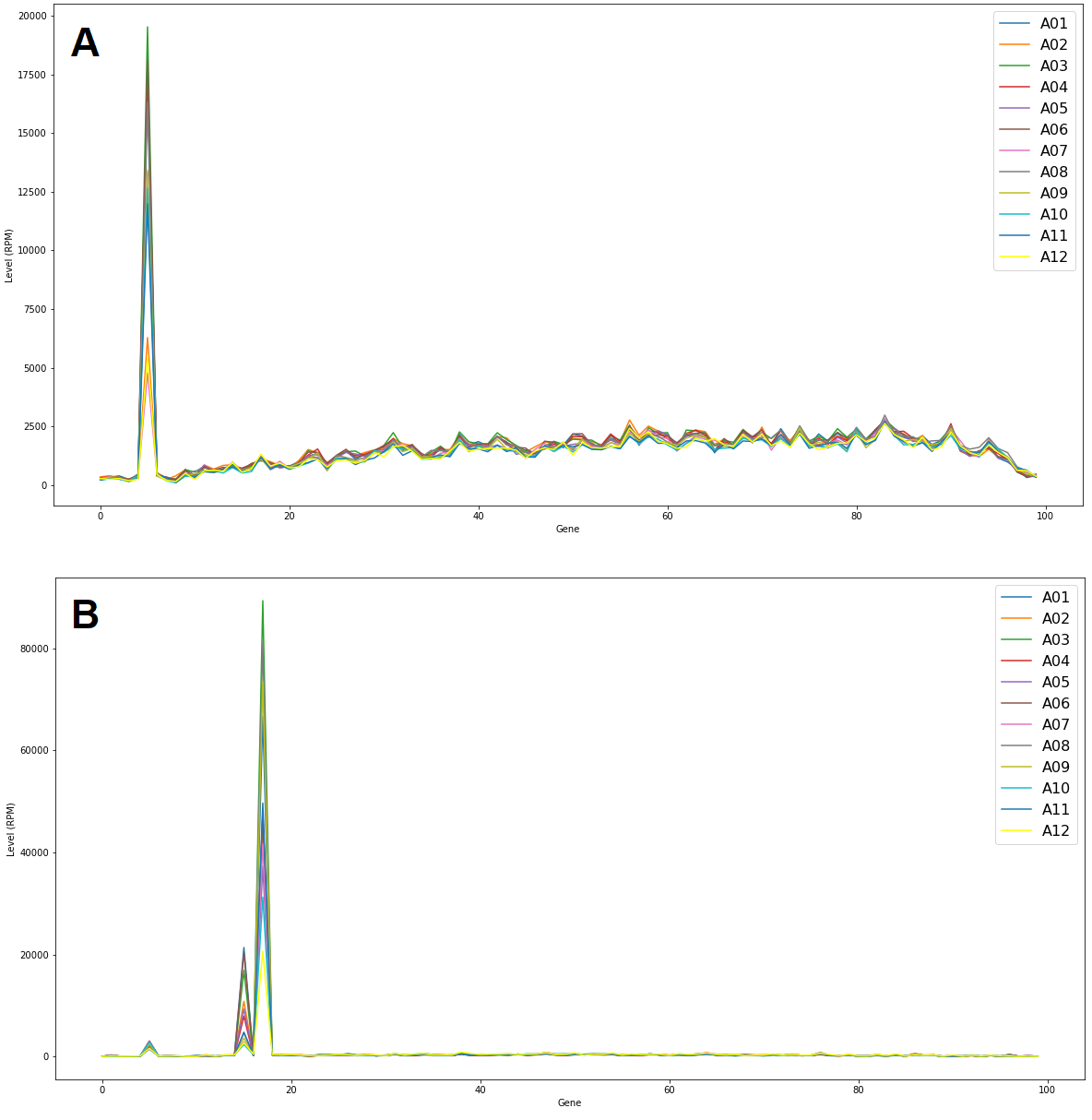

Supplement: S4 Fig — The X axis represents the relative position in the gene (i.e. 20 means 20% of the initial positions of the gene). A: analysis for CCGG sites; B: analysis for CCNGG sites. Legend: A01: FST-R1; A02: FST-R2; A03: FST-R3; A04: FNC-R1; A05: FNC-R2; A06: FNC-R3; A07: DST-R1; A08: DST-R2; A09: DST-R3; A10: DNC-R1; A11: DNC-R2; A12: DNC-R3; R1, R2 and R3: biological triplicates; FST - BRS Formosa Stressed Treatment; FNC – BRS Formosa Negative Control; DST – BRS Dourada Stressed Treatment; DNC – BRS Dourada Negative Control. (TIF) [file pone.0296254.s004.tif]

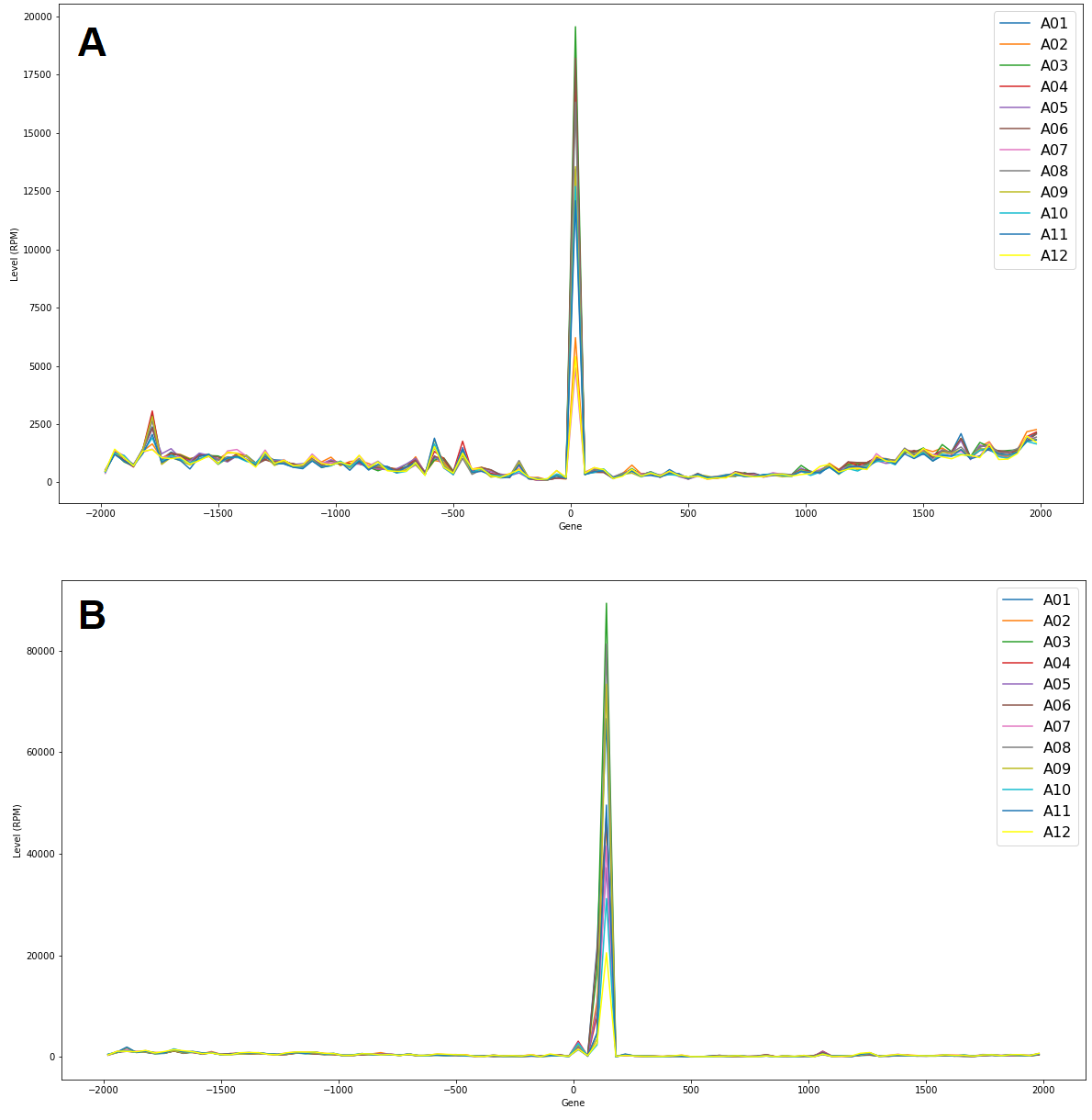

Supplement: S5 Fig — The x-axis represents the 2000 bp window before and after the start of transcription. Graph A represents the analysis for CCGG site; B represents analysis for CCNGG site. A01: FST-R1; A02: FST-R2; A03: FST-R3; A04: FNC-R1; A05: FNC-R2; A06: FNC-R3; A07: DST-R1; A08: DST-R2; A09: DST-R3; A10: DNC-R1; A11: DNC-R2; A12: DNC-R3; R1, R2 and R3: biological triplicates; FST - BRS Formosa Stressed Treatment; FNC – BRS Formosa Negative Control; DST – BRS Dourada Stressed Treatment; DNC – BRS Dourada Negative Control. (TIF) [file pone.0296254.s005.tif]

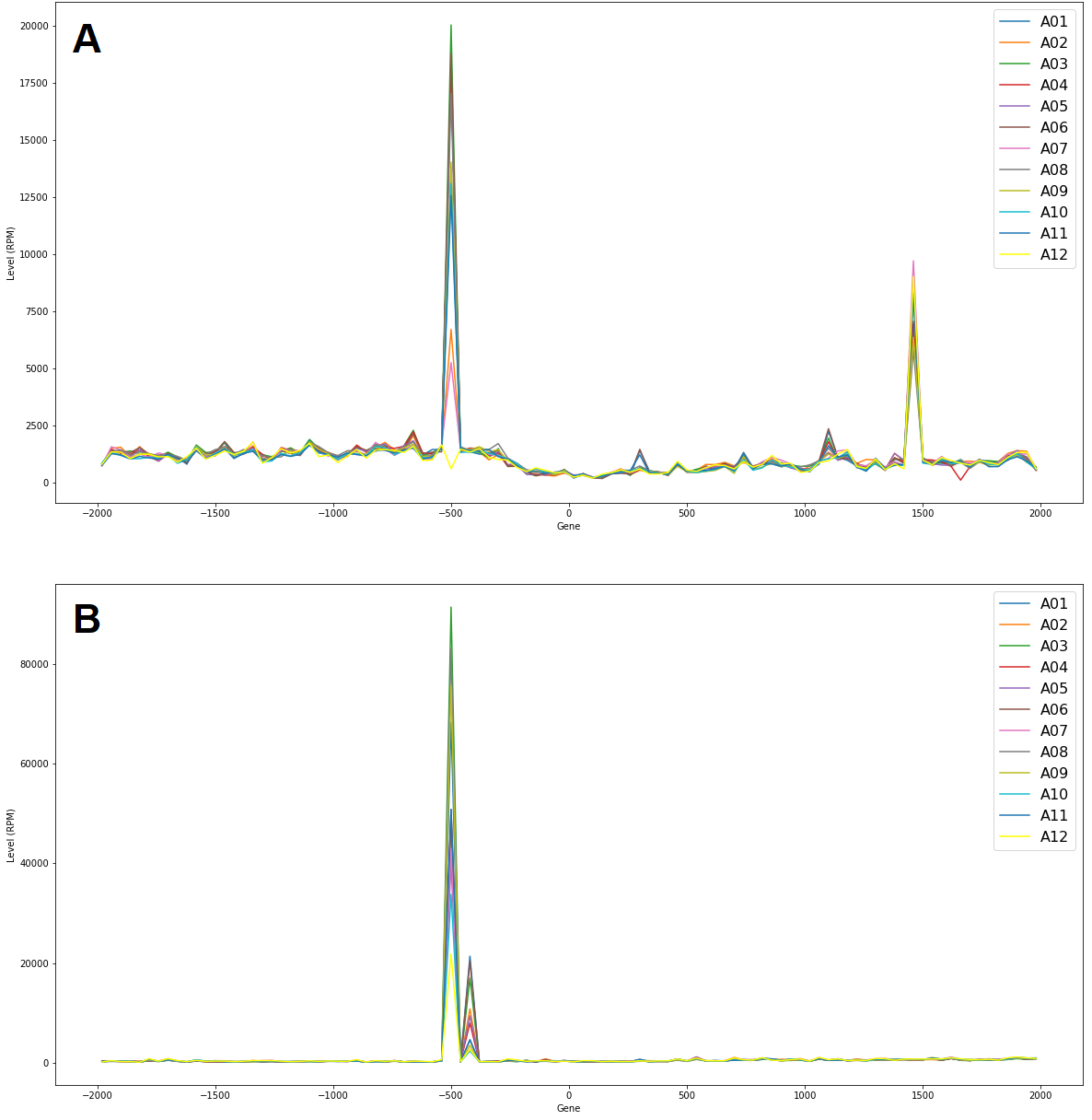

Supplement: S6 Fig — The x-axis represents the 2000 bp window before and after the start of transcription. Graph A represents the analysis for CCGG site; B represents analysis for CCNGG site A01: FST-R1; A02: FST-R2; A03: FST-R3; A04: FNC-R1; A05: FNC-R2; A06: FNC-R3; A07: DST-R1; A08: DST-R2; A09: DST-R3; A10: DNC-R1; A11: DNC-R2; A12: DNC-R3; R1, R2 and R3: biological triplicates; FST - BRS Formosa Stressed Treatment; FNC – BRS Formosa Negative Control; DST – BRS Dourada Stressed Treatment; DNC – BRS Dourada Negative Control. (TIF) [file pone.0296254.s006.tif]

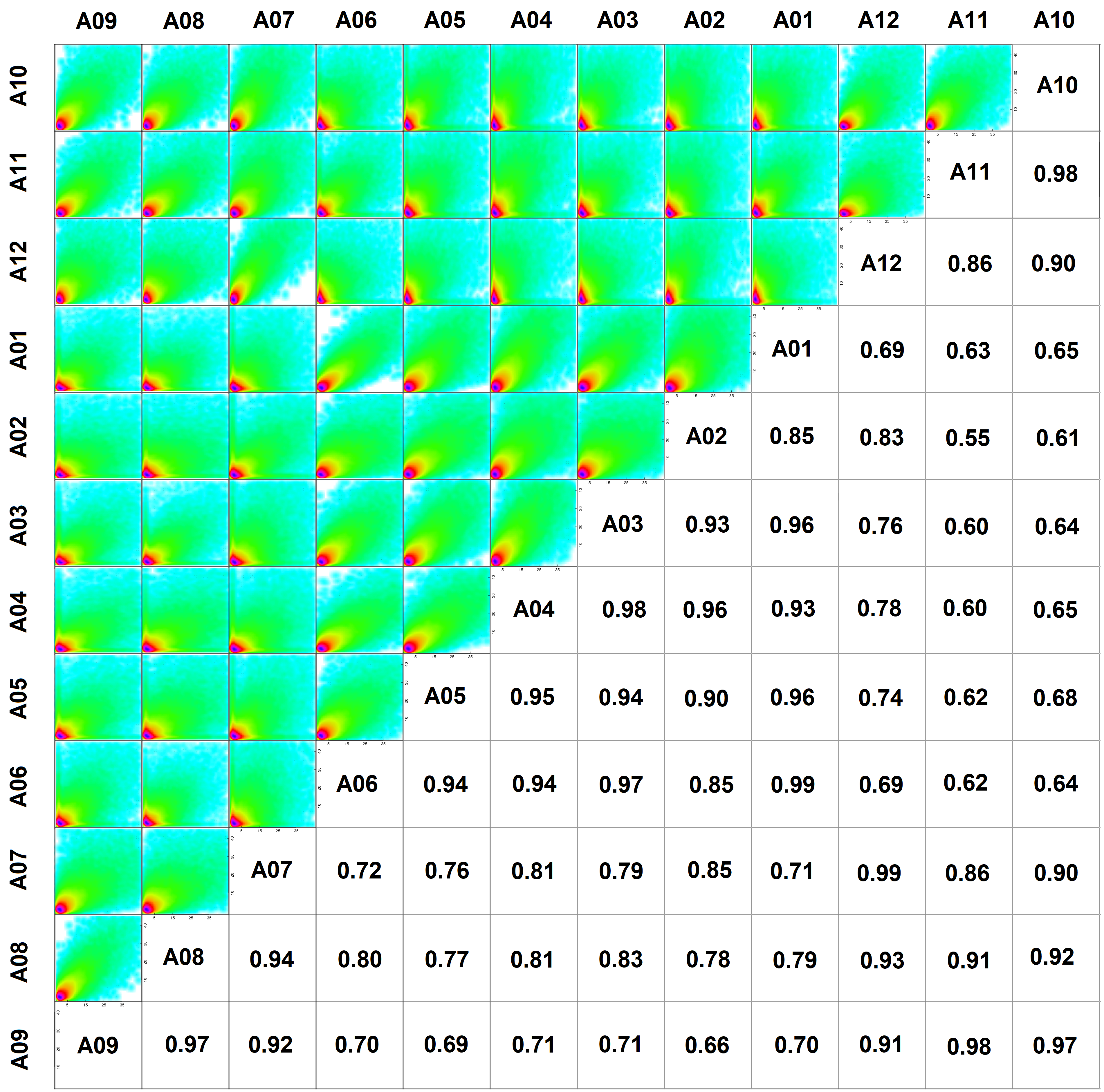

Supplement: S7 Fig — The upper left triangle is the scatterplot of the methylation level between two samples that are diagonally across the image, the lower right triangular region is the corresponding Pearson correlation coefficient. A01: FST-R1; A02: FST-R2; A03: FST-R3; A04: FNC-R1; A05: FNC-R2; A06: FNC-R3; A07: DST-R1; A08: DST-R2; A09: DST-R3; A10: DNC-R1; A11: DNC-R2; A12: DNC-R3; R1, R2 and R3: biological triplicates; FST - BRS Formosa Stressed Treatment; FNC – BRS Formosa Negative Control; DST – BRS Dourada Stressed Treatment; DNC – BRS Dourada Negative Control. (TIF) [file pone.0296254.s007.tif]

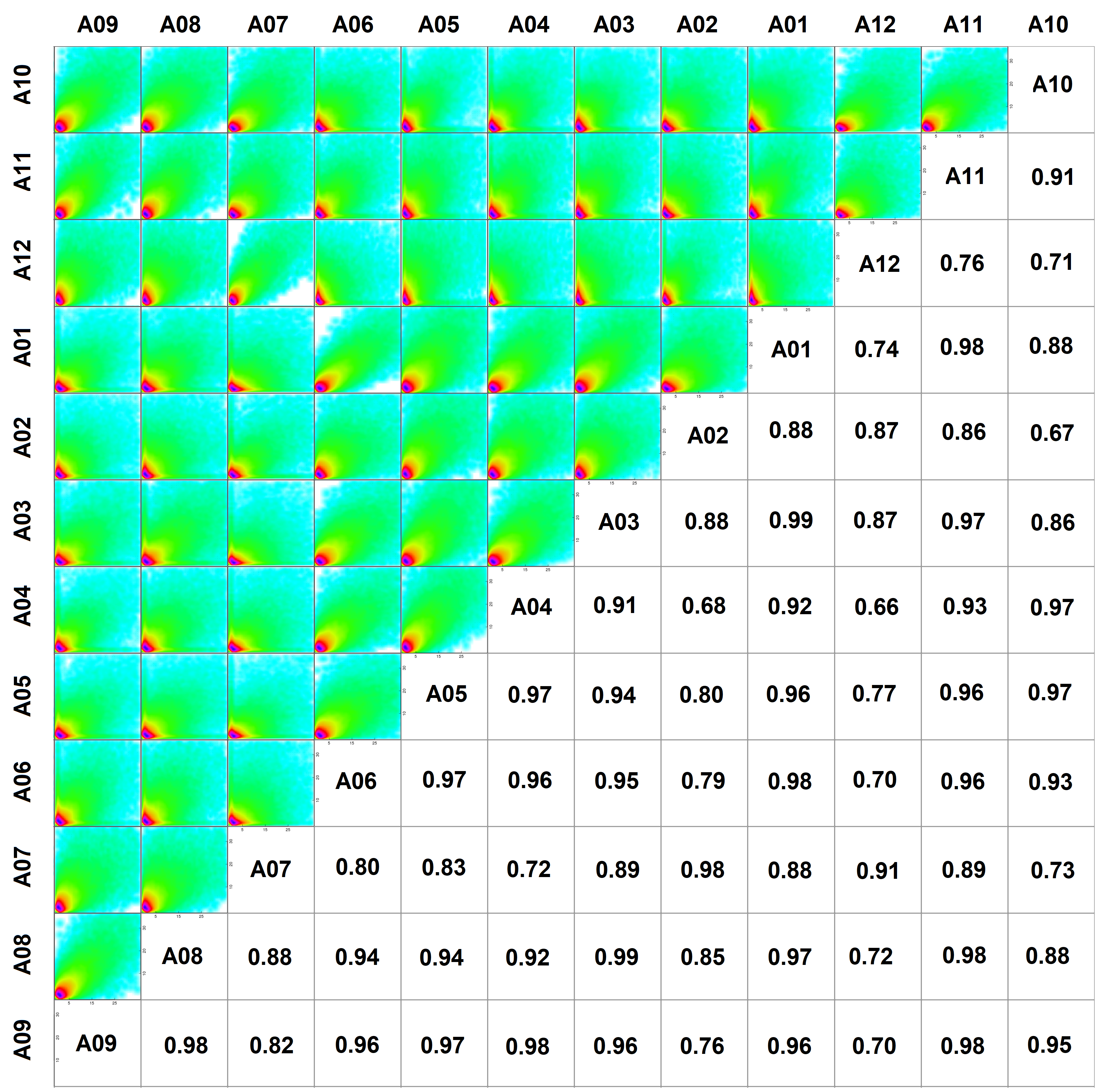

Supplement: S8 Fig — The upper left triangle is the scatterplot of the methylation level between two samples that are diagonally across the image, the lower right triangular region is the corresponding Pearson correlation coefficient. A01: FST-R1; A02: FST-R2; A03: FST-R3; A04: FNC-R1; A05: FNC-R2; A06: FNC-R3; A07: DST-R1; A08: DST-R2; A09: DST-R3; A10: DNC-R1; A11: DNC-R2; A12: DNC-R3; R1, R2 and R3: biological triplicates; FST - BRS Formosa Stressed Treatment; FNC – BRS Formosa Negative Control; DST – BRS Dourada Stressed Treatment; DNC – BRS Dourada Negative Control. (TIF) [file pone.0296254.s008.tif]

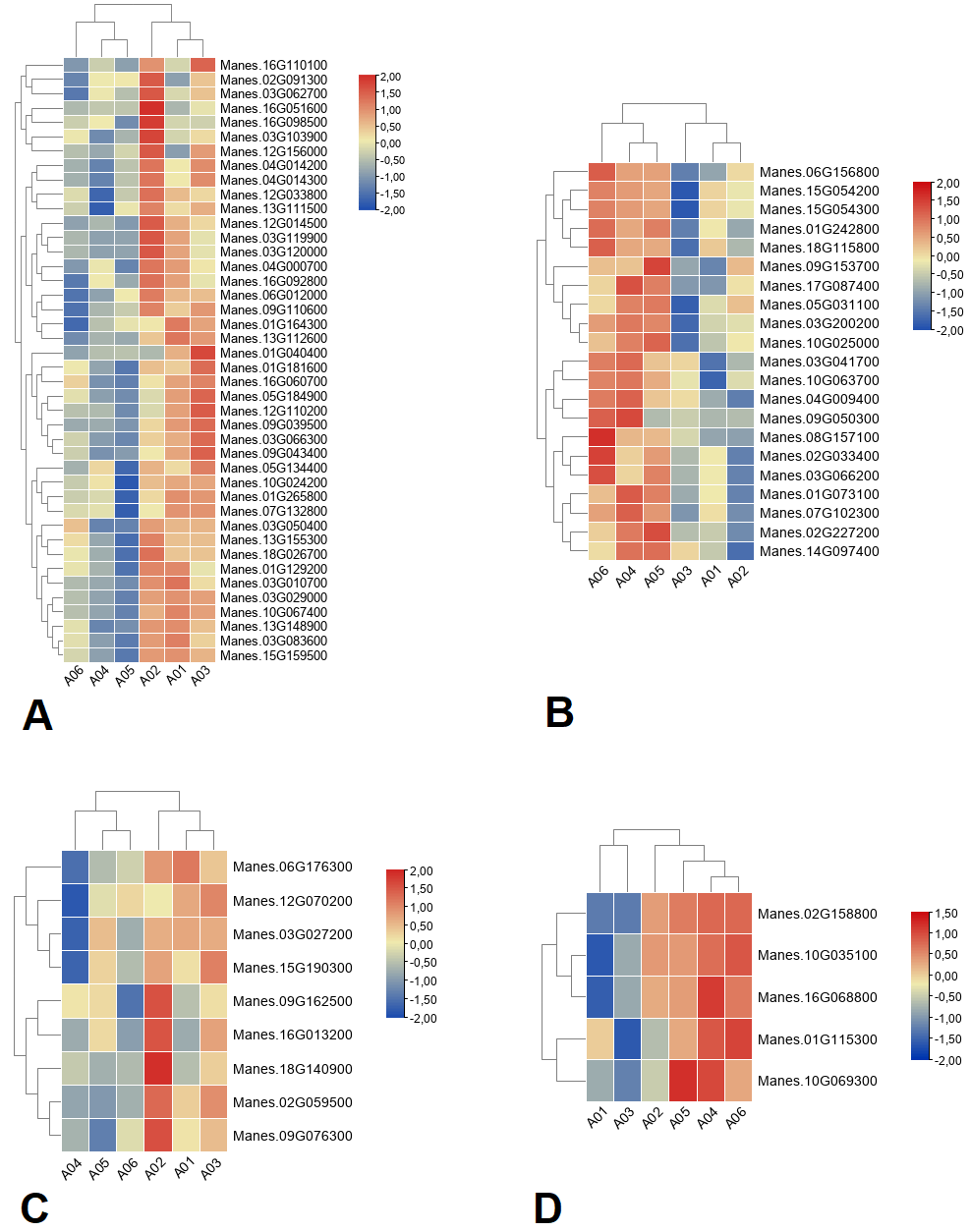

Supplement: S9 Fig — Heatmap graph of methylated genes (p-value ≤ 0.05 and log2FC > 1) in BRS Formosa variety, presented by the biological replicas (R1, R2 and R3) in drought condition or control without stress, based on log2 of RPM; A01 (FST-R1); A02 (FST-R2); A03 (FST-R3); A04 (FNC-R1); A05 (FNC-R2); A06 (FNC-R3); FST – BRS Formosa Stressed Treatment, FNC – BRS Formosa Negative Control; A) hypermethylated genes in CCGG sites; B) hypomethylated genes in CCGG sites; C) hypermethylated genes in CCNGG sites; D) hypomethylated genes in CCNGG sites. (TIF) [file pone.0296254.s009.tif]

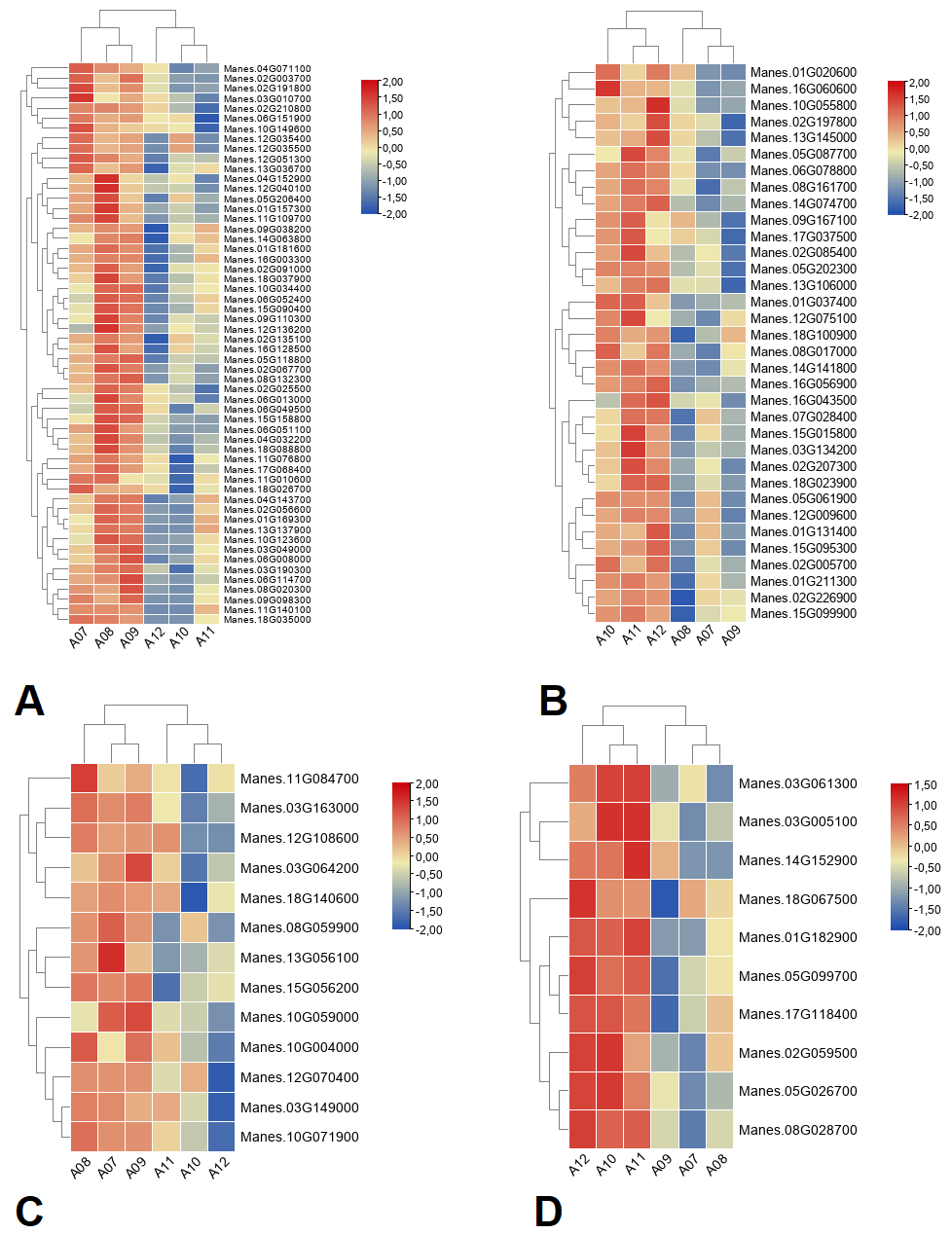

Supplement: S10 Fig — Heatmap graph of methylated genes (p-value ≤ 0.05 and log2FC > 1) in BRS Dourada variety, presented by the biological replicas (R1, R2 and R3) in drought condition or control without stress, based on log2 of RPM; A07 (DST-R1); A08 (DST-R2); A09 (DST-R3); A10 (DNC-R1); A11 (DNC-R2); A12 (DNC-R3); DST – BRS Dourada Stressed Treatment, DNC – BRS Dourada Negative Control; A) hypermethylated genes in CCGG sites; B) hypomethylated genes in CCGG sites; C) hypermethylated genes in CCNGG sites; D) hypomethylated genes in CCNGG sites. (TIF) [file pone.0296254.s010.tif]
