# Supplementary material for: Exploiting DNA methylation in cassava under water deficit for crop improvement
Source: PLoS One. 2024 Feb 22;19(2):e0296254. doi: 10.1371/journal.pone.0296254 (PMC10883565; doi:10.1371/journal.pone.0296254)
Supplement: Attachment — Submitted filename: Silva Filho et al_PlosOne_2023 responses.docx [file pone.0296254.s020.docx]

**Response reviewers**

**Journal requirements:**

2. In your Data Availability statement, you have not specified where the minimal data set underlying the results described in your manuscript can be found. PLOS defines a study's minimal data set as the underlying data used to reach the conclusions drawn in the manuscript and any additional data required to replicate the reported study findings in their entirety. All PLOS journals require that the minimal data set be made fully available. For more information about our data policy, please see <http://journals.plos.org/plosone/s/data-availability>.

**Response**: All data sets are available at the link <<https://doi.org/10.6084/m9.figshare.21330708.v2>>, which is provided in the 'Data Availability Statement' section.

3. We note that you have included the phrase “data not shown” in your manuscript. Unfortunately, this does not meet our data sharing requirements. PLOS does not permit references to inaccessible data. We require that authors provide all relevant data within the paper, Supporting Information files, or in an acceptable, public repository. Please add a citation to support this phrase or upload the data that corresponds with these findings to a stable repository (such as Figshare or Dryad) and provide and URLs, DOIs, or accession numbers that may be used to access these data. Or, if the data are not a core part of the research being presented in your study, we ask that you remove the phrase that refers to these data.

**Response**: We have revised our text. All the information is available in the manuscript and the link provided in the 'Data Availability Statement' section

4. We note that Figure 2 in your submission contain copyrighted images. All PLOS content is published under the Creative Commons Attribution License (CC BY 4.0), which means that the manuscript, images, and Supporting Information files will be freely available online, and any third party is permitted to access, download, copy, distribute, and use these materials in any way, even commercially, with proper attribution. For more information, see our copyright guidelines: <http://journals.plos.org/plosone/s/licenses-and-copyright>.

**Response**: Figure 2 does not contain copyrighted images. The authors themselves have provided this figure, which will now be included in the respective document.

**Reviewer #1:**

The paper is interesting however some issues need to be clarified, especially in the materials and methods section.

1. Apart form the photos of the plants after the completion of drought stress (with claer differences in morphology) and the Transpirable Soil Water (FTSW) calculation, no other morphological or physiological measurements are described. Can you provide some more data regarding the differences in morphology and physiology og the 2 genotypes after drought stress?

**Response**: Additional information was added at the beginning of the 'Results' section. Please let us know if you have any remaining questions.

Materials and methods section needs to be divided into sections, first describing more clearly the experimental design and the duration of the drought stress and then focusing on the molecular studies.

**Response**: **Response**: We appreciate your feedback. Acknowledging your comment, we recognize that restructuring our methodology into sections enhanced readability and understanding. We have corrected this aspect accordingly

2. Conclusion section needs to be rewritten in some parts and need to give more realistic outcomes.

**Response**: Thank you for the suggestion regarding the conclusion of our manuscript. We've restructured it to be more concise and direct, while also incorporating additional relevant information about our obtained results.

3. please see the attachment and provide answers accordingly.

**Response**: Ok, thank you.

**Reviewer #2:**

The study is particularly important and original. The methodology followed is detailed, with many analyzes and interpretations of the results. I want just a few clarifications on the methodology. What were the prevailing conditions in the greenhouse, in which the experiment was carried out?

**Response**: Additional information has been included within the 'Materials and Methods' section to address these queries.

Why were only 5 plants per variety selected?

**Response**: There were 5 plants per plot. Please review the modifications within the 'Materials and Methods' section.

Why are only the parents of the variety BRS Formosa listed and not both varieties.

**Response**: Please check the following sentence in the text.

“BRS Dourada is a local variety with an unknown pedigree, specifically developed for fresh consumption.”
